# The role of F_1 _ATP synthase beta subunit in WSSV infection in the shrimp, *Litopenaeus vannamei*

**DOI:** 10.1186/1743-422X-7-144

**Published:** 2010-06-30

**Authors:** Yan Liang, Jun-Jun Cheng, Bing Yang, Jie Huang

**Affiliations:** 1 Key Laboratory of Sustainable Utilization of Marine Fisheries Resources, the Ministry of Agriculture; Yellow Sea Fisheries Research Institute, Chinese Academy of Fishery Sciences, Qingdao 266071, China

## Abstract

**Background:**

Knowledge of the virus-host cell interaction could inform us of the molecular pathways exploited by the virus. Studies on viral attachment proteins (VAPs) and candidate receptor proteins involved in WSSV infection, allow a better understanding of how these proteins interact in the viral life cycle. In this study, our aim was to find some host cellular membrane proteins that could bind with white spot syndrome virus (WSSV).

**Results:**

Two proteins were evident by using a virus overlay protein binding assay (VOPBA) with WSSV. A protein with molecular weight 53 kDa, named BP53, was analyzed in this study, which was homologous with the F_1_-ATP synthase beta subunit by mass spectrometry analysis. Rapid amplification of cDNA ends (RACE) PCR was performed to identify the full-length cDNA of the *bp53 *gene. The resulting full-length gene consisted of 1836 bp, encoding 525 amino acids with a calculated molecular mass of 55.98 kDa. The deduced amino acid sequence contained three conserved domains of the F_1_-ATP synthase beta subunit. BP53 was therefore designated the F_1_-ATP synthase beta subunit of *L. vannamei*. The binding of WSSV to BP53 were also confirmed by competitive ELISA binding assay and co-immunoprecipitation on magnetic beads. To investigate the function of BP53 in WSSV infection, it was mixed with WSSV before the mixture was injected intramuscularly into shrimp. The resulting mortality curves showed that recombinant (r) BP53 could attenuate WSSV infection.

**Conclusions:**

The results revealed that BP53 is involved in WSSV infection. Here is the first time showed the role of shrimp F_1_-ATP synthase beta subunit in WSSV infection.

## Background

White Spot Syndrome Virus (WSSV) is a species in the newly described genus *Whispovirus*, in the family *Nimaviridae*. It is one of the most devastating viral pathogens of shrimp farming, causing high mortality and considerable economic loss. WSSV is an enveloped virus with a large, double stranded, circular genome (~300 kb). The complete genome sequence has been described from three WSSV isolates and it has at present the largest animal virus genome known [1,2]. A total of 531 putative ORFs were identified by sequence analysis, among which 181 ORFs are likely to encode functional proteins [1]. Among 181 ORFs, the proteins encoded by 18 ORFs show 40 to 68% identity to known proteins from other viruses or organisms or contain an identifiable functional domain. And the proteins encoded by 133 ORFs were with no homology to any known proteins or motifs [1]. For this reason, WSSV has still to be fully characterized.

The interactions of viral proteins with host cell membranes are important for viruses to enter into host cells, replicate their genome, and produce progeny particles [3,4]. Some structural proteins of WSSV, such as VP26, VP28, VP37 (VP281), VP466 and VP68, have been reported to interact with host cell components, so as to significantly delay or neutralize WSSV infection [5-11]. To enter the host cell, a virus needs to bind to a receptor, and sometimes a co-receptor, before being able to deliver its genome. PmRab7 (*Penaeus monodon *Rab7) appears to be one specific shrimp protein that can interact with VP28, and is the first to be identified as one that binds directly to a major viral envelope protein of WSSV [8]. Studies on viral attachment proteins (VAPs) and candidate receptor proteins involved in WSSV infection, allow a better understanding of how these proteins interact in the viral life cycle. Knowledge of the virus-host cell interaction could inform us of the molecular pathways exploited by the virus, and also provides further targets that could be pursued for antiviral drug development.

Although considerable progress has been made in the molecular characterization of WSSV, a little information on shrimp genes which are involved in WSSV infection are known. In this article, to find out the host cellular membrane proteins that can bind with WSSV, virus overlay protein binding assay (VOPBA) and co-immunoprecipitation on magnetic beads were conducted. We investigated the interaction of F_1_-ATP synthase beta subunit with WSSV, and for the first time describe the role of F_1_-ATP synthase beta subunit during WSSV infection.

## Results

### A 53 kDa shrimp protein binds to WSSV by VOPBA

**Figure 1:**
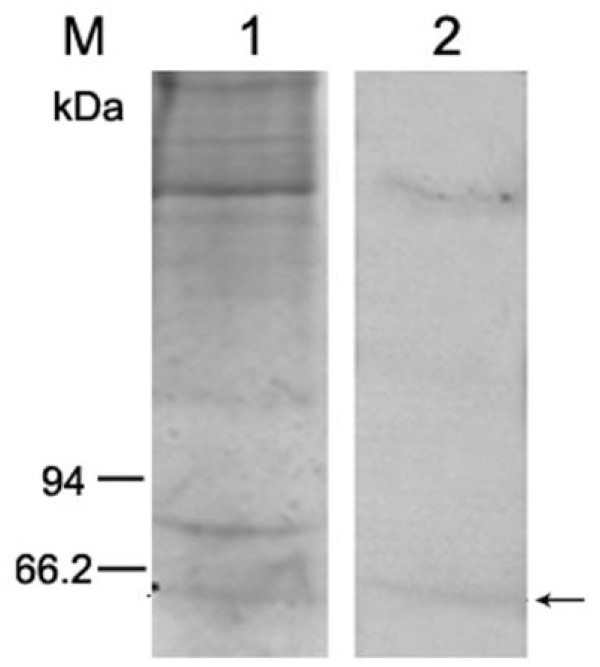
**Results of VOPBA to bind with WSSV**. Lane 1, Coomassie blue stained gel of CMP without incubated with DIG-WSSV. Lane 2, blot of CMP incubated with DIG-labeled WSSV. The arrow indicates a binding protein with a molecular mass of 53 kDa.

Virus overlay protein binding assay (VOPBA) is a standard technique to identify cell molecules involved in virus binding. To identify WSSV binding proteins from the cell-surface of shrimp gills, the VOPBA was carried out. Two distinct protein bands from gill cellular membrane protein (CMP) were revealed using SDS-PAGE. One band had an estimated molecular mass about 200 kDa, and the other with a molecular mass of 53 kDa (Fig. 1). The latter 53-kDa WSSV-binding band (BP53) was extracted from an SDS-12% polyacrylamide gel for MALDI (matrix assisted laser desorption/ionization)-TOF combined mass spectrometry (MS) analysis.

A BLASTP search of the results against the GenBank database http://www.ncbi.nlm.nih.gov showed that BP53 resembles the F_1_-ATP synthase beta subunit of *Drosophila melanogaster*, with ten matching peptides (Table 1).

**Table 1:** Results of BP53 mass spectrometry analysis compared to the best-matched database protein

Protein Name	Accession No.	Protein MW	Protein PI	Pep. Count	Protein Score
ATP synthase beta subunit [Drosophila melanogaster]	gi:287945	53487.1	5.19	10	365

**Peptide Information**					

**Calc. Mass**	**Observ. Mass**	**Start Seq.**	**End Seq.**	**Sequence**	**Ion Score**

975.5621	975.614	174	184	IGLFGGAGVGK	

1191.6731	1191.6415	395	404	GVQKILQDYK	

1367.7528	1367.8346	116	127	IINVIGEPIDER	75

1406.681	1406.7665	198	211	AHGGYSVFAGVGER	99

1435.7539	1435.8387	283	296	FTQAGSEVSALLGR	69

1439.7892	1439.8732	254	266	VALTGLTVAEYFR	

1457.8396	1457.8735	185	197	TVLIMELINNVAK	

1677.9281	1678.0283	67	81	LVLEVAQHLGENTVR	75

1921.9653	1922.0756	267	282	DQEGQDVLLFIDNIFR	

2252.0686	2252.1958	297	317	IPSAVGYQPTLATDMGSMQER	

### Full length cDNA of *bp53 *and motif analysis

**Figure 2:**
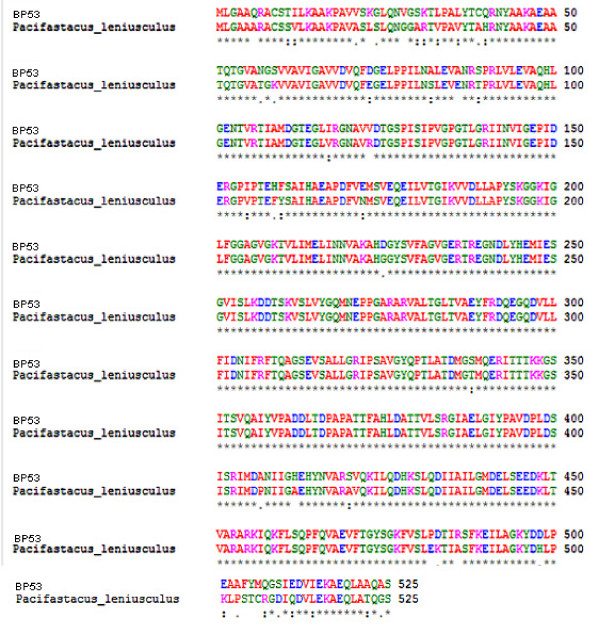
**Amino acid sequence alignment between BP53 and freshwater crayfish (*Pacifastacus leniusculus*)**. The sequence was showed in single-letter abbreviations of amino acid.

To obtain the 5'- and 3'-end sequences of *bp*53, rapid amplification of cDNA ends (RACE) PCR was carried out. The full-length cDNA of *bp53 *was generated, which consisted of 1836 bp with an open reading frame (ORF) of 1578 bp encoding 525 deduced amino acids (GenBank, EU401720). There was a 5' non-coding sequence of 20 bp and 3 conserved domains including F_1 _ATP synthase beta subunit nucleotide-binding domain, ATP synthase alpha/beta chain N terminal domain, ATP synthase alpha/beta chain C terminal domain according to the NCBI Conserved Domain Database website. This indicated that the deduced protein was a shrimp F_1_-ATP synthase beta subunit. Three well-conserved regions of the F_1_-ATP synthase beta subunit were found including the Walker motif A (GGAGVGKT), the DELSEED motif, and the ATPase_alpha_beta signature domain (PAVDPLDSIS). A homology search against GenBank using BLAST, showed 91% similarity with the F_1_-ATP synthase beta subunit of the crayfish *Pacifastacus leniusculus *(Fig. 2).

### Binding between rBP53 and WSSV is specific

**Figure 3:**
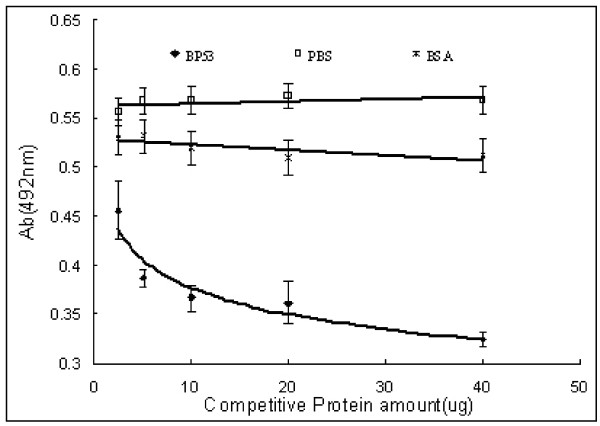
**Compete ELISA binding assay**. Graph showing decreasing absorbance that resulted when increasing rBP53 was added to compete with CMP in the ELISA assay for WSSV binding activity. Error bars indicate standard deviations.

We have developed competitive ELISA binding tests to determine the specificity of BP53 binding to WSSV particles. ELISA tests with WSSV particles against CMP, purified rBP53 and BSA (control), showed that the binding between CMP and WSSV could be inhibited by rBP53, and that the inhibition was dose dependent (Fig. 3). No competitive binding was observed between BSA or PBS and WSSV. Here results showed that the binding between rBP53 and WSSV is specific.

**Figure 4:**
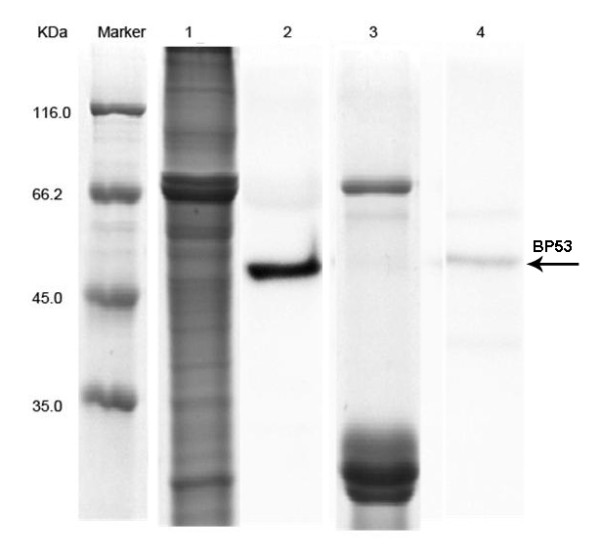
**Coupling immunomagnetic separation on magnetic beads with western blot for detection of the interaction between BP53 and WSSV**. Line marker, pre-stained protein molecular mass markers (MBI, USA); Line 1, SDA-PAGE of shrimp gill CMPs; Line 3, SDS-PAGE of the eluted components on dynabeads coated with WSSV particles after flowed with shrimp gill CMPs; Line 2 and 4, identification of BP53 using anti-rBP53 antibody by western blot. The samples loaded in Line 2 was shrimp gill membrane proteins, as same as Line 1; The samples loaded in Line 4 was the eluted components on dynabeads coated with WSSV particles after flowed with shrimp gill membrane proteins, as same as Line 3.

To confirm the specific interaction between BP53 in shrimp gill CMPs with WSSV, the co-immunoprecipitation on magnetic beads was performed. The eluted proteins that could bind with WSSV were separated by SDS-PAGE, which contained several bands. After a western blot with anti-rBP53 antibody showed the existence of BP53 with an approximately 56 kDa molecular weight in the eluted proteins (Fig.4). The extraction of gill CMPs were used as control, in which a same band was specifically detected by anti-rBP53 antibody (Fig. 4). As shown in the results above, BP53 was one of the binding proteins against WSSV.

### Innoculum preincubation with rBP53 delayed mortality from WSSV challenge

**Figure 5:**
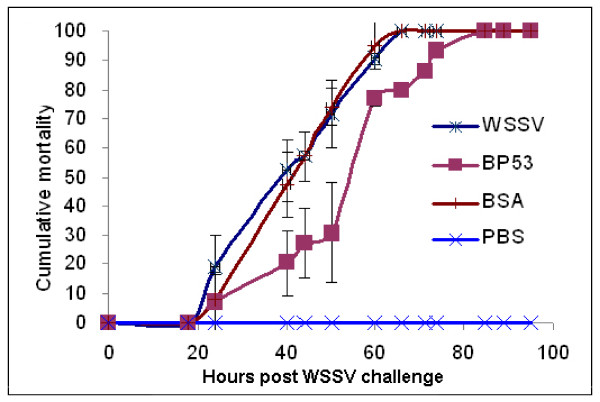
**Neutralization of WSSV with rBP53**. At 0 hour, shrimp were injected as follows: group 1, WSSV alone (3000 virions ml^-1^/shrimp); group 2, PBS buffer; group 3, WSSV preincubated with rBP53; group 4, WSSV plus BSA. Cumulative mortality data represent the pooled results for three replications (*n *= 20 for each group). Error bars indicate standard deviations.

To identify whether BP53 play roles in involving WSSV infection, the neutralization experiment was carried out on shrimp. Shrimp mortality increased steadily from 20 h, and reached to 100% at 66 h for both groups injected with WSSV alone (positive control) and groups injected with WSSV pre-incubated with BSA (non-specific protein control) (Fig. 5). By contrast, there was no shrimp mortality in the PBS buffer-injected group (negative control group) (Fig. 5). The mortality levels in groups injected with WSSV pre-incubated with rBP53 were lower from 24 h to 74 h when compared to the positive control, which reach to 100% at 85 h after challenged. The results indicated that pre-incubation with rBP53 could delay shrimp death from WSSV challenge.

## Discussion

The virus overlay technique used here has previously been employed to identify a number of putative receptor proteins [12-15]. While the technique is normally undertaken with reduced and denatured proteins separated by SDS polyacrylamide gel electrophoresis, the successful identification of a number of receptors would suggest that a degree of protein renaturation occurs during the overlay process. Following VOPBA without renaturation of protein after SDS-PAGE, the binding activity of CMP was lost, and no bands were revealed (data not shown). However, when SDS-PAGE-separated CMPs were transferred to a PVDF membrane and renaturized before incubated with DIG-virus, their binding activity was restored. In this report, one of the protein with molecular weight 53 kDa, BP53, was identified, which has the deduced amino acid sequence be highly similar to that of the F_1_-ATP synthase beta subunit of *Pacifastacus leniusculus *[16].

Recently, an interferon-like protein (IntlP) homologue was identified for the first time in *Penaeus *(*Marsupenaeus*) *japonicus *shrimp, where it plays an important role in antiviral activities [17] and has some similarity to an F_0_-ATP synthase beta chain [18,19]. A comparative proteomic analysis was used to analyze differentially expressed proteins in virus-infected shrimp, *P. mondon*, by Wang *et al*. [20] and Bourchookarn *et al*. [21]. In their results the ATP synthase beta subunit was significantly up-regulated when shrimp were infected with WSSV or YHV. All the reports above suggest that ATP synthase of shrimp plays an important role in antiviral defense against both WSSV and YHV.

For enveloped viruses, *in vivo *neutralization experiments are routinely conducted to study the function of viral envelope proteins and to identify viral protein epitopes involved in the virus infection process. This might lead to the development of preventive approaches for virus disease control such as blocking the host-virus binding site to prevent the viral entry into host cells. Of the WSSV envelope proteins identified, VP28 was found to be involved in systemic shrimp infection that could be blocked by VP28 polyclonal antiserum [22]. Using an alternative strategy for the first time in shrimp, Sritunyalaksana et al [8]showed that administration of the host VP28-Binding protein PmRab7 ( or an antibody against it ) could reduce and delay mortality upon subsequent WSSV challenge. Here we have shown with similar experiments that administration of BP53 could also delay mortality caused by WSSV. The results suggested that F_1_-ATP synthase beta subunit plays a role in the WSSV infection.

## Conclusions

F_1_F_0_-ATP synthase complexes play a central role in the synthesis of ATP in all living organisms, which was originally described from the inner membrane of mitochondria. It was found also on the surface of human umbilical vein endothelial cells (HUVECs) where it served as a receptor for angiostatin [23]. Previous reports suggested that the F_1 _portion of ATP synthase resides on the cell surface where it may serve as a cell membrane receptor [24]. While the mitochondrial synthase utilizes the proton gradient generated by oxidative phosphorylation to power ATP synthesis, the cell surface synthase has instead been implicated in numerous other activities, including the mediation of intracellular pH, cellular response to antiangiogenic agents and cholesterol homeostasis [25]. BP53 was found to exist on the cell surface of both gill and hemocyte cells by indirect immno-fluorescence assays and Immune colloidal gold techniques (unpublished), confirming that surface F_1_-ATP synthase beta subunit exists in shrimp. Interestingly, F_1_-ATP synthase beta subunit is identified to serve as the receptor for the invertebrate prokineticin, astakine, and it is located on the plasma membrane of crayfish Hpt cells [26].It will be interesting to further investigate the precise role of F_1_-ATP synthase beta subunit binding to WSSV in the host infection process, and its related chain reactions.

## Materials and methods

### Shrimp

A batch of shrimp (400), *Litopenaeus vannamei*, approximately 6 - 8 g (fresh weight) and 6 - 8 cm long, were purchased from a shrimp farm in Qingdao, Shandong Province, China, and cultured in 80 l tanks (at 25 °C) filled with sea water circulated by air pumps. The shrimp were randomly sampled and tested by PCR for absence of WSSV and used for neutralization tests, and some used for preparation of cellular membrane proteins (CMPs).

### WSSV purification and DIG labeled

The intact WSSV viral particles from infected crayfish tissues were purified as described by Xie et al [27]. The optical density of the purified virion samples was measured at 600 nm wavelength using spectrophotometer then the virion concentration was caculated according to the formula as described in Zhou et al [28].

To prepare DIG-labeled virus for VOPBA and ELISA binding test, the virion was incubated with DIG-NHS for 2 h at room temperature at the molar reaction ratio 1:70. DIG labeled components were isolated from the reaction mixture through a Sephedax G25 column. The resulting suspension was measured for protein concentration by the Bradford method [28] and stored at -75°C in 50 μl aliquots.

### Preparation of cellular membrane protein

The CMP extracts were prepared as previous described [5]. In brief, gill tissue was homogenized in a Dounce homogenizer with 5 times volume of ice-cold RSB-NP40 (containing: MgCl_2_, 1.5 mM; Tris-HCl, 10 mM; NaCl, 10 mM; NP-40, 1%; EDTA, 2 mM; and 0.5 mM PMSF; 0.7 μg ml^-1 ^pepstatin; leupeptin to 5 μg ml ^-1 ^leupeptin; and 5 μg ml^-1 ^chymostatin; which were freshly added). After centrifugation at 600 ×g and 800 ×g for 10 min respectively to remove nuclei, debris, and chromosomes, the membrane components in the supernatant were pelleted by centrifuging at 100,000 ×g for 20 min at 4°C. The resulting suspension was measured for protein concentration by the Bradford method [29] and stored at -75°C in 50 μl aliquots.

### Determination of binding proteins by VOPBA

To identify shrimp membrane proteins involved in WSSV binding, a VOPBA was carried out. A total of 50 μg CMPs per lane were separated on 12% SDS-PAGE gel and transferred 80 min at 280 mA to PVDF membrane. The transferred proteins were renatured following the modified method as described in Kameshita *et al *[30]. In brief, the SDS was removed by washing the membrane with 30 ml 20 mM Tris-HCl (pH 8.0) containing 20% isopropanol for 20 min twice. Then the membrane washed by 30 ml Buffer A (20 mM Tris-HCl, 4 mM 2-mercaptoethanol, pH 8.0) for 20 min twice. Followed twice washing by Buffer A containing 6 M guanidine HCl for 15 min, then renatured the transferred proteins with five changes of 30 ml Buffer A containing 0.03% Tween 20. After renaturation, the membrane was blocked with 5% skim milk in PBS at 37°C for 1 h. A total 800 μg DIG-WSSV in 1% skim milk in PBS was incubated with the membrane overnight at 4°C. After three washes with PBS contained 0.05% Tween 20, the membrane was incubated with 1:2000 Anti-Digoxigenin-AP (Roche, Germany) at 37°C for 2 h. After wash, the signal was generated by BCIP/NBT substrate kit (Picere, USA). The corresponding binding protein was cutted from a 12% SDS-PAGE gel for mass spectrometry analysis (MS).

### RACE cloning of *bp53 *gene

Rapid amplification of cDNA ends (RACE) of *bp53 *gene was performed. Total RNA was extracted from the hemolymph using TRI Reagent (Invitrogen) following the manufacturer's instructions. RNA (2 μg) was reverse-transcribed with an oligo (dT) primer using M-MLV reverse transcriptase at 42°C for 1 h, and then at 70°C for 15 min to obtain cDNA.

The PCR reaction to obtain the 3' end of *bp53 *cDNA was performed according to the 3'-Full RACE Core Set (TaKaRa) protocol. Five specific sense primers were designed, based on the sequence of the clones obtained above (Table 1). The reverse sense primer was (Oligo dT-3sites Adaptor Primer): 5'-CTG ATC TAG AGG TAC CGG ATC C-3'. The fragment obtained was then cloned into a PMD-18T vector (Tiangen, China) and sequenced using an ABI377 Automated Sequencer (Applied Biosystems).

Two specific reverse primers (primer 6 and primer 7, Table 2) were designed based on the 3' RACE sequences obtained in order to clone the 5' end of *bp53 *cDNA. Nested-PCR amplification was performed to obtain the 5' end of BP53 using the sense primer adaptor dG (5'-CTA CTA CTA CTA GGC CAC GCG TCG ACT AGT ACG GGG GGG GGG GGG GGG-3') and the two reverse primers (primer 6 and primer 7). The purified PCR product was ligated with PMD-18T vector (Tiangen), and three of the positive clones were sequenced on an ABI 377 Automated Sequencer (Applied Biosystems).

**Table 2:** Specific primers for BP53 RACE

Specific sense primers for 3' RACE	Sequence
Primer 1	5'-CTG AGG TAC CGG ATC CCG TGT CGC CCT GAC TGG T-3'

Primer 2	5'-CTG AGG TAC CGG ATC CCA ACA TTT TCC GCT TCA CA-3'

Primer 3	5'-CTG AGG TAC CGG ATC CCC CTG ACT GGT CTG ACT GTG G-3'

Primer 4	5'-CTG AGG TAC CGG ATC CGA AGG TCA AGA TGT GCT GCT C-3'

Primer 5	5'-CTG AGG TAC CGG ATC CGA CAA CAT TTT CCG CTT CAC A-3

Specific reverse primers for 5' RACE	Sequence

Primer 6	5'-AGA GCA GCA CAT CTT GAC CTT CC-3'

Primer 7	5'-ACA GTC AGA CCA GTC AGG GCG ACA-3'

### Recombinant BP53 expression

The entire protein-coding region (525 amino acids) of *bp53 *cDNA was amplified using PCR and two synthetic primers (5'-ATG CTC GAG TCT CCT CCG CCA GG-3';, forward primer containing a Xho I restriction enzyme site; 5'-ATT AAG CTT ACG CTG GCC TGG GCA-3', reverse primer containing a Hind III restriction enzyme site. The amplified PCR product was digested with Hind III and Xho I, separated on a 1% agarose gel and purified from the gel using a gel extraction kit (Qiagen). Purified DNA was ligated to a pBAD-gIIIA vector (Qiagen) in-frame with a sequence encoding six histidine residues at the N-terminus. The resulting recombinant plasmid, pBAD-gIIIA/BP53, was transformed into the host *E. coli *TOP10. Induced by L-arabinose, the protein was expressed in the form of inclusion bodies.

### Purification and renaturation of rBP53

The insoluble His-tagged fusion protein was first purified as inclusion bodies. After dissolving the inclusion bodies in 6 mol l^-1 ^guanidine hydrochloride, further purification of the protein was carried out using a Ni-NTA agarose kit (Qiagen) according to the manufacturer's protocol. The total amount of purified protein was quantified by the Bradford method using BSA as the standard and its purity was checked using 12% SDS-PAGE. The eluted protein was then refolded by dialyzing for 12 h against buffer (50 mM NaCl, 1 mM EDTA, 10% glycerol, 1% glycine, 20 mM phosphate, pH7.4) containing respectively 4 M urea, 2 M urea and 0 M urea separately.

### Co-immunoprecipitation on magnetic beads

Dynabeads M-280 tosylactivated (Invitrogen) were chosen to capture the interacted proteins of shrimp gill CMP against WSSV. 10 μg dynabeads coated with purified WSSV particles were prepared according to manufacturer' instructions. For conjugation of WSSV to the tosylactivated beads, the beads were washed twice in buffer A (0.1 M borate buffer, pH 9.5) and conjugation was carried out for 24 h at room temperature with vortex. Conjugation solution contained at most 200 μg WSSV particles diluting in final volume of 150 μl buffer A, and 100 μl buffer C (3M ammonium sulphate in buffer A). At the end of the conjugation procedure, removed supernatant by place the tube on a magnet, which would allow the beads to pellet completely. After 1 hour blocking in 1 ml buffer D (PBS with 0.5% (wt/vol) BSA) at 37°C, beads were washed three times with buffer E (PBS with 0.1% (wt/vol) BSA) and equilibrated in this buffer (480 μl). 400 μg shrimp gill membrane proteins were mixed with the WSSV coupled beads by vortex and incubated at RT for 1 h to capture the target protein. Discard the supernatant, the beads were washed three times with PBS buffer (pH 7.4) and then boiled in 20 μl SDS-PAGE buffer for 5 min to elute target protein. The eluted products were subjected to 12% SDS-PAGE, followed the western bolt assay. 1:1000 dilution of rabbit anti-rBP53 antibody was used to identify the binding proteins, which incubated at 37°C for 2 h. Then 1:2000 anti-rabbit HRP antibody was used as secondary antibody, which incubated at 37°C for 1 h. After thoroughly washing, the color was developed with SuperSignal West Pico Chemiluminescent Substrate (Pierce).

### Determination of binding specificity by competitive ELISA binding assay

Flat-bottomed 96-well ELISA plates (costar) were coated with 2 μg CMP at 4°C overnight and then blocked with 5% non-fat milk in PBS buffer for 2 h at 37°C. The plates were washed three times with PBS buffer containing 0.05% Tween 20, following which DIG labeled virus, were added and incubated with either 2.5 μg, 5 μg, 10 μg, 20 μg and 40 μg rBP53 for 1 h at 37°C. The virus incubated with 40 μg BSA/PBS was used as a control. After 1 h incubation at 37 °C, and three washes, 1:2000 Anti-Digoxigenin-POD (Roche) was added. Finally the reaction was visualized using the HRP substrate O-phenylenediamine, and stopped by the addition of 2 M H_2_SO_4_. The absorbance was immediately read at 492 nm using a TECAN SAFIRE (Fluorescence, Absorbance and Luminescence) Reader.

### *In vivo *neutralization assay

This in vivo assay was developed to test whether BP53 could block WSSV infection in shrimp. Purified and renaturized rBP53 (0.4 mg ml^-1 ^in PBS, pH 7.5) was incubated with WSSV (3000 virions ml^-1^, final concentration) [26] for 1 h at room temperature. Then the mixture was injected intramuscularly into shrimp in the lateral area of the fourth abdominal segment at 0.1 ml per shrimp using a 1 ml sterile syringe. WSSV alone was used as a positive control. WSSV was pre-incubated with bovine serum albumin (BSA, 0.4 mg/ml, in PBS, pH 7.5) to evaluate the effect of the same protein concentration on WSSV infection. Shrimp injected with PBS, pH 7.5, were regarded as a negative control. Each treatment was replicated with three batches of 20 shrimp. Shrimp mortality was monitored daily, and deceased shrimp were examined for the presence of WSSV by dot-blot hybridization.

## Competing interests

The authors declare that they have no competing interests.

## Authors' contributions

YL carried out all the experiments, acquisition of experimental data and drafted the manuscript. JJC participated in the in vivo neutralization test and co-immunoprecipitation on magnetic beads. BY participated in the work of obtain the 3'-end sequence of *bp*53. JH involved in design of the study and helped to revise the manuscript. All authors read and approved the final manuscript.
